# Antitumor immunity of low-dose cyclophosphamide: changes in T cells and cytokines TGF-beta and IL-10 in mice with colon-cancer liver metastasis

**DOI:** 10.1093/gastro/goz060

**Published:** 2019-12-05

**Authors:** Xiao-Ming Huang, Nan-Rong Zhang, Xu-Tao Lin, Cai-Yan Zhu, Yi-Feng Zou, Xiao-Jian Wu, Xiao-Sheng He, Xiao-Wen He, Yun-Le Wan, Ping Lan

**Affiliations:** 1 Department of Hepatobiliary Surgery, The Sixth Affiliated Hospital of Sun Yat-sen University, Guangzhou, Guangdong, P. R. China; 2 Guangdong Institute of Gastroenterology; Guangdong Provincial Key Laboratory of Colorectal and Pelvic Floor Diseases, The Sixth Affiliated Hospital of Sun Yat-sen University, Guangzhou, Guangdong, P. R. China; 3 Department of Anesthesiology, The Sixth Affiliated Hospital of Sun Yat-sen University, Guangzhou, Guangdong, P. R. China; 4 Department of Gastrointestinal Endoscopy, Department of Colorectal Surgery, The Sixth Affiliated Hospital of Sun Yat-sen University, Guangzhou, Guangdong, P. R. China; 5 Department of Pharmacy, The Sixth Affiliated Hospital of Sun Yat-sen University, Guangzhou, Guangdong, P. R. China; 6 Department of Colorectal Surgery, The Sixth Affiliated Hospital of Sun Yat-sen University, Guangzhou, Guangdong, P. R. China

**Keywords:** colon cancer, liver metastasis, cyclophosphamide, immune microenvironment

## Abstract

**Background:**

The tumor immune microenvironment is one of the most important prognostic factors in liver metastasis from colorectal cancer. Low-dose cyclophosphamide (CTX) is widely believed to be involved in the modulation of the immune system. However, the underlying mechanism of low-dose CTX remains unknown. This study aimed to investigate the antitumor immunity of low-dose CTX in the treatment of colon-cancer liver metastasis.

**Methods:**

Thirty mice were randomly divided into five groups. After liver metastasis was established in colon-cancer models, mice in the treatment groups were injected with low-dose CTX (20 mg/kg) at different time points. Liver and spleen tissues were examined for T-cell markers via flow cytometry. Interleukin (IL)-10 and transforming growth factor (TGF)-β1 expression levels in liver tissues were analysed by immunohistochemistry. Serum interferon (IFN)-γ and IL-10 levels were detected by enzyme-linked immunosorbent assay. An additional 20 mice were randomly allocated into two groups and the survival times were recorded.

**Results:**

The expression levels of CD4^+^ T cells, CD8^+^ T cells, and IFN-γ were down-regulated, whereas those of IL-10 and TGF-β1 were up-regulated in liver metastasis from colon cancer in mice. Furthermore, the local and systemic microenvironments of the liver were altered, which led to reduced antitumor immune responses and subsequently liver metastasis. However, treatment with low-dose CTX reversed these effects. The survival times of mice treated with low-dose CTX were significantly longer than those of the other groups.

**Conclusions:**

Low-dose CTX exerts its antitumor activity by changing the systemic and local immune microenvironments and enhancing immune regulation in mice. CTX could be used as a drug to prevent and treat liver metastasis from colon cancer.

## Introduction

Colorectal cancer is the third most common cancer in men and the second in women worldwide. Approximately 50% of the patients with stage III and 20% with stage II colorectal cancers will develop liver metastases [1, 2]. Currently, surgery is the only therapeutic option with curative potential for colorectal liver metastasis [3]. Although early-stage colorectal cancer is often curable by surgery and has a 5-year survival rate of approximately 90%, the prognosis for patients with metastatic colorectal cancer remains poor, with 5-year survival rates in the range of 10%–20% [4]. Recent study showed that perioperative chemotherapy could reduce the risk of recurrence after resection of colorectal liver metastases [5]. However, the development of drug resistance in tumor cells is a significant problem [6]. Therefore, additional therapeutic approaches for the elimination of resistant tumor cells are required.

A healthy immune system is believed to be crucial for the control of cancer because it may protect against nascent cancers by destroying malignant cells before their development into detectable tumors [7]. Consequently, an appealing alternative therapeutic strategy involves stimulation of the immune system to induce a potent antitumor response. The chemotherapeutic agent cyclophosphamide (CTX) is a nitrogen mustard alkylating agent that is generally considered to be an immunosuppressive drug. CTX is not active *in vitro* and is hydrolysed to aldehyde phosphoramide by liver P450 enzymes and transported into tissues to form active phosphoramide mustard. As a well-known broad-spectrum antitumor and immunosuppressant drug, CTX is widely used to treat various types of cancer and autoimmune diseases. As an antitumor drug, it is often combined with other antitumor drugs and used, for example, to treat malignant lymphoma, breast cancer, small-cell lung cancer, neuroblastoma, acute leukemia, and chronic lymphocytic leukemia, and its synergistic effects have been reported [8–12].

However, there is evidence that CTX may have different immunomodulatory effects at different doses [13]. High-dose CTX inhibits the production of inflammatory cells and inflammatory factors. Conversely, low-dose CTX enhances the immune response against various tumor antigens by suppressing regulatory T cells (Treg cells) and down-regulating interleukin (IL)-10 [14–18]. Studies have indicated that the immunostimulatory effect of CTX plays a role in the selective depletion of CD4^+^CD25^+^ Treg cells in both experimental and human tumors [19–21]. CTX at a low dose (20 mg/kg) has been shown to augment host immune responses, such as suppression of CD4^+^CD25^+^ Treg cells, down-regulation of T-cell-derived IL-10 expression, and production of transforming growth factor (TGF)-β, which are widely believed to play crucial roles in immune tolerance [22]. Although low-dose CTX is believed to be involved in modulating the immune system, an optimal administration regimen has not been fully elucidated. In this study, we compared the effects of low-dose CTX at different time points on the expression of the anti-inflammatory cytokines IL-10 and TGF-β1, T-cell subsets including CD4^+^CD25^+^Foxp3^+^ T cells, and tumor immunity in mice.

## Materials and methods

### Animals

Female Balb/c mice, 6–8 weeks old (mean body weight, 20 g), were purchased from the Experimental Animal Center of Sun Yat-sen University (Guangzhou, China) and housed in cages (six to each cage) under specific pathogen-free conditions. All mice received humane care according to protocols approved by the University’s Animal Care Committee and in compliance with the guidelines on animal welfare of the National Committee for Animal Experiments. This study was approved by the Ethics Committee of the Sixth Affiliated Hospital of Sun Yat-sen University.

### Cell line

CT26 cells were purchased from the American Type Culture Collection (CRL-2638; Manassas, VA, USA), maintained in RPMI-1640 medium with 10% fetal calf serum, and cultured in a 37°C humidified atmosphere of 5% CO_2_.

### Drugs

CTX (Sigma-Aldrich, St Louis, MO, USA) was dissolved in phosphate-buffered saline (PBS) to a concentration of 20 mg/mL and diluted to a concentration of 2 mg/mL immediately prior to use.

### Surgical procedure and CTX dosing

Mice (*n* = 30) were randomly divided into five groups. Liver-metastasis models of colon cancer were established in the experimental groups (CLM-CTX1 [colon-cancer liver metastasis-cyclophosphamide 1], CLM-CTX2, CLM-CTX3, CLM). The normal group was used as the negative control group. The surgical procedure for splenic tumor-cell injection has been described extensively [22]. Tumor cells (5 × 10^4^ cells) in 50 μL PBS were injected into the spleen using a 32-G needle in the experimental groups (CLM-CTX1, CLM-CTX2, CLM-CTX3, CLM). Mice in the negative control group (normal group) received a splenic injection of 50 μL PBS solution without tumor cells (*n* = 6). Mice in the first treatment group (CLM-CTX1; *n* = 6) received intraperitoneal (IP) injections with CTX (20 mg/kg) post operation (Day 0), whereas those in the second (CLM-CTX2; *n* = 6) and third treatment groups (CLM-CTX3; *n* = 6) were given IP injections of CTX (20 mg/kg) on Days 0 and 7 after operation and on Days 0, 7, and 14 after operation, respectively. The mice in the CLM group were not injected with CTX. All mice were sacrificed on Day 21 post operation. An additional 20 female Balb/c mice were randomly allocated into two groups, namely the CLM and CLM-CTX groups, to study survival time. Mice in the CLM-CTX group were injected with low-dose CTX into the abdomen on the day establishing liver metastasis of colon cancer, while the CLM-group mice were injected with the same amount of saline. The survival times were recorded and a survival curve was plotted.

### Cell preparation and flow-cytometry analysis

Single-cell suspensions were prepared from the spleens and livers of mice obtained at Day 21 post surgery. Red blood cells were lysed with 0.17 mol/L ammonium chloride, washed with PBS, and counted using a Cellometer^®^ Auto T4 cell counter (Nexcelom Bioscience, Lawrence, MA, USA). Cells (1 × 10^6^) were then stained with anti-CD4, anti-CD25, anti-Foxp3, and anti-CD8 antibodies according to the manufacturer’s instructions. All antibodies were purchased from eBiosciences (San Diego, CA, USA). The numbers and percentages of the T-cell subsets were determined using a FACSCanto^™^ flow cytometer (BD Biosciences, Franklin Lakes, NJ, USA). Data were collected from 10,000 cells and analysed using CellQuest software (BD Pharmingen, San Jose, CA, USA).

### Histological and immunohistochemical analysis

The livers and spleens of mice were surgically removed, paraffin-embedded, and sectioned (4 μm). Hematoxylin and eosin (HE) staining was performed to assess the histopathological condition of the tissues and confirm liver metastasis. The localization of IL-10 (Beijing Biosynthesis Biotechnology, Beijing, China) and TGF-β1 (Bioworld Technology Inc., St Louis Park, MN, USA) was visualized by immunohistochemical staining as previously described [22]. Tissue sections (4 μm) were prepared from formalin-fixed, paraffin-embedded tissues. After deparaffinization and rehydration for antigen retrieval, slides were heated in 10 mM citrate buffer (pH 6.0) in a microwave oven or pressure cooker and cooled to room temperature.

### Image acquisition and rendering

Bright field images of the HE and antibody-stained sections were visualized under a Leica DMI4000 B microscope (Leica Microsystems, Wetzlar, Germany) and photomicrographs were taken at ×400 magnification using a digital image-acquisition system.

### Quantification of staining

Positive staining was identified when the cells or stroma showed clear brown staining, quantified by measuring the integrated optical density (IOD) in three representative areas per section, and expressed as a percentage of the average number of positive sections/total area. All the staining data were analysed using ImageJ 1.48 u software (National Institutes of Health, Bethesda, USA).

### Estimation of IL-10 and interferon-γ expression by enzyme-linked immunosorbent assay

The levels of IL-10 and interferon (IFN)-γ in sera from mice were estimated using an enzyme-linked immunosorbent assay kit (eBiosciences). All tests were performed in triplicate. Absorbance at 450 nm was determined using a microplate reader (iMark™; Bio-Rad Laboratories, Hercules, CA, USA).

### Statistical analysis

All data are expressed as the mean ± standard deviation. Data were analysed by analysis of variance (ANOVA) using SPSS16.0 software (SPSS Inc., Chicago, IL, USA). The differences between groups were analysed using one-way ANOVA and the least significant difference test. *P*-values <0.05 were considered statistically significant.

## Results 

### Induction of liver metastases by splenic injection of CT26 colon-cancer cells

**Figure 1.**
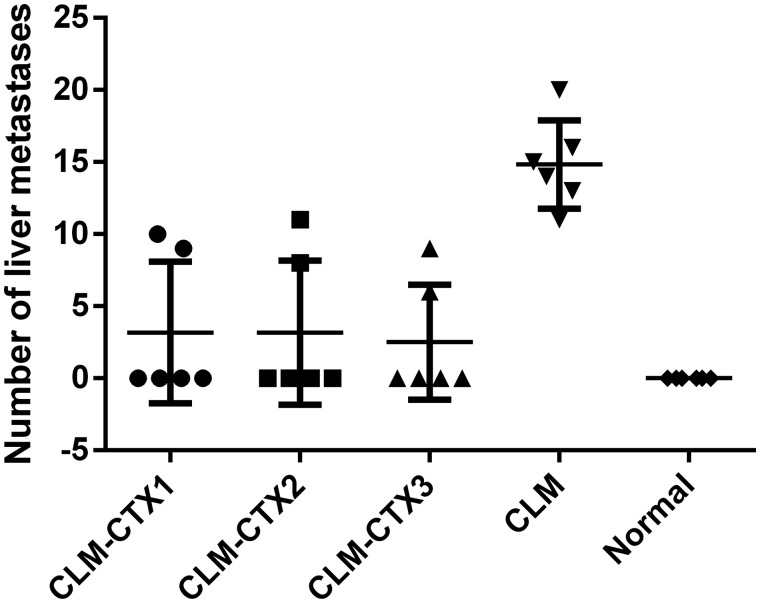
The number of mice that developed liver metastasis of colon cancer through splenic tumor-cell injection (5 × 10^4^ CT26 cells) in different treatment groups. The liver metastasis of colon cancer was observed only in two of six mice in the CLM-CTX1, CLM-CTX2, and CLM-CTX3 groups. Macroscopic liver metastasis of colon cancer was observed in all mice in the CLM group. No macroscopic and microscopic liver metastases were observed in the normal group. Mice in the CLM-CTX1 group (*n* = 6) were intraperitoneally injected with CTX (20 mg/kg) post surgery immediately. Mice in the CLM-CTX2 group (*n* = 6) were intraperitoneally injected with CTX (20 mg/kg) on Days 0 and 7 post surgery. Mice in the CLM-CTX3 group (*n* = 6) were intraperitoneally injected with CTX (20 mg/kg) on Days 0, 7, and 14 post surgery. The mice of the liver metastasis only (CLM) group (*n* = 6) were not injected with CTX. CLM, colon-cancer liver metastasis; CLM-CTX, colon-cancer liver metastasis-cyclophosphamide; CTX, cyclophosphamide.

**Figure 2.**
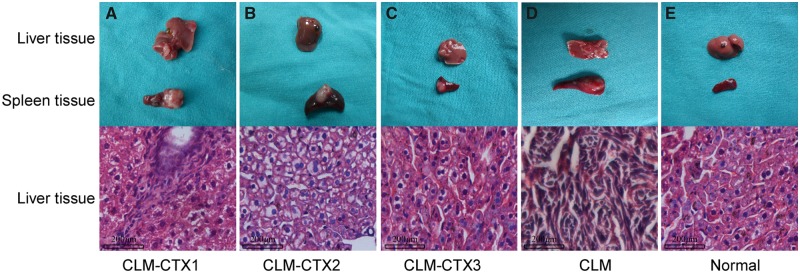
Establishment of macroscopic and microscopic liver metastases through splenic tumor-cell injection (5 × 10^4^ CT26 cells) in different treatment groups. The liver metastasis of colon cancer was observed only in two of six mice in the CLM-CTX1, CLM-CTX2, and CLM-CTX3 groups. Macroscopic liver metastasis of colon cancer was observed in all mice in the CLM group. No macroscopic and microscopic liver metastases were observed in the normal group. Mice in the CLM-CTX1 group (*n* = 6) were intraperitoneally injected with CTX (20 mg/kg) post surgery immediately. Mice in the CLM-CTX2 group (*n* = 6) were intraperitoneally injected with CTX (20 mg/kg) on Days 0 and 7 post surgery. Mice in the CLM-CTX3 group (*n* = 6) were intraperitoneally injected with CTX (20 mg/kg) on Days 0, 7, and 14 post surgery. The mice of the liver-metastasis-only (CLM) group (*n* = 6) were not injected with CTX. CLM, colon-cancer liver metastasis; CLM-CTX, colon-cancer liver metastasis-cyclophosphamide; CTX, cyclophosphamide.

All mice that survived the injection of 5 × 10^4^ CT26 cells also survived for 21 days after surgery and were sacrificed on that day. In the CLM-CTX1, CLM-CTX2, and CLM-CTX3 groups, liver metastases of colon cancer were observed in two of six mice in each group. All mice in the CLM group showed more macroscopic liver metastases than those in the treatment groups. No macroscopic or microscopic liver metastases were observed in the normal group (Figures 1 and 2).

### Flow-cytometry analysis of the CD4^+^CD25^+^FOXP3^+^ Treg-cell population

**Figure 3.**
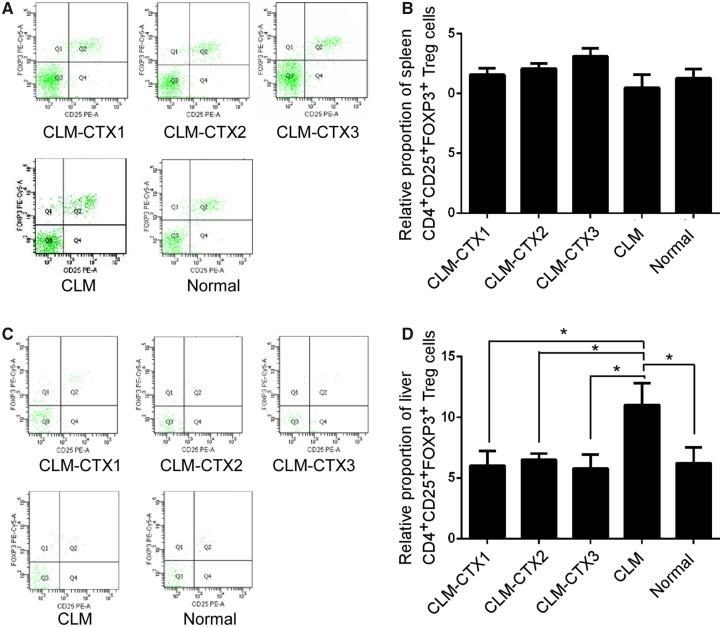
Flow-cytometry analysis of the CD4^+^CD25^+^FOXP3^+^ Treg-cell population in the spleen and liver. In the spleen, no significant differences were observed among the five groups with respect to the proportion of CD4^+^CD25^+^FOXP3^+^ Treg cells in the total CD4^+^ T-cell population (both *P* > 0.05) ((**A**) and (**B**)). In the liver, the proportion of CD4^+^CD25^+^FOXP3^+^ Treg cells in the total CD4^+^ T-cell population was significantly higher in the CLM group than in other groups (*P* = 0.032) ((**C**) and (**D**)). No significant differences were observed among the other four groups (both *P* > 0.05). Mice in the CLM-CTX1 group (*n* = 6) were intraperitoneally injected with CTX (20 mg/kg) post surgery immediately. Mice in the CLM-CTX2 group (*n* = 6) were intraperitoneally injected with CTX (20 mg/kg) on Days 0 and 7 post surgery. Mice in the CLM-CTX3 group (*n* = 6) were intraperitoneally injected with CTX (20 mg/kg) on Days 0, 7, and 14 post surgery. The mice of the liver-metastasis-only (CLM) group (*n* = 6) were not injected with CTX. CLM, colon-cancer liver metastasis; CLM-CTX, colon-cancer liver metastasis-cyclophosphamide; CTX, cyclophosphamide.

In the present study, there were no significant differences among the five groups with respect to the proportion of CD4^+^CD25^+^FOXP3^+^ Treg cells in the splenic CD4^+^ T-cell population (both *P* > 0.05) (Figure 3A and B). In the liver, the proportion of CD4^+^CD25^+^FOXP3^+^ Treg cells in the total CD4^+^ T-cell population was significantly higher in the CLM group than in the other groups (*P* = 0.032) (Figure 3C and D).

### Flow-cytometry analysis of the CD8^+^ T-cell population

**Figure 4.**
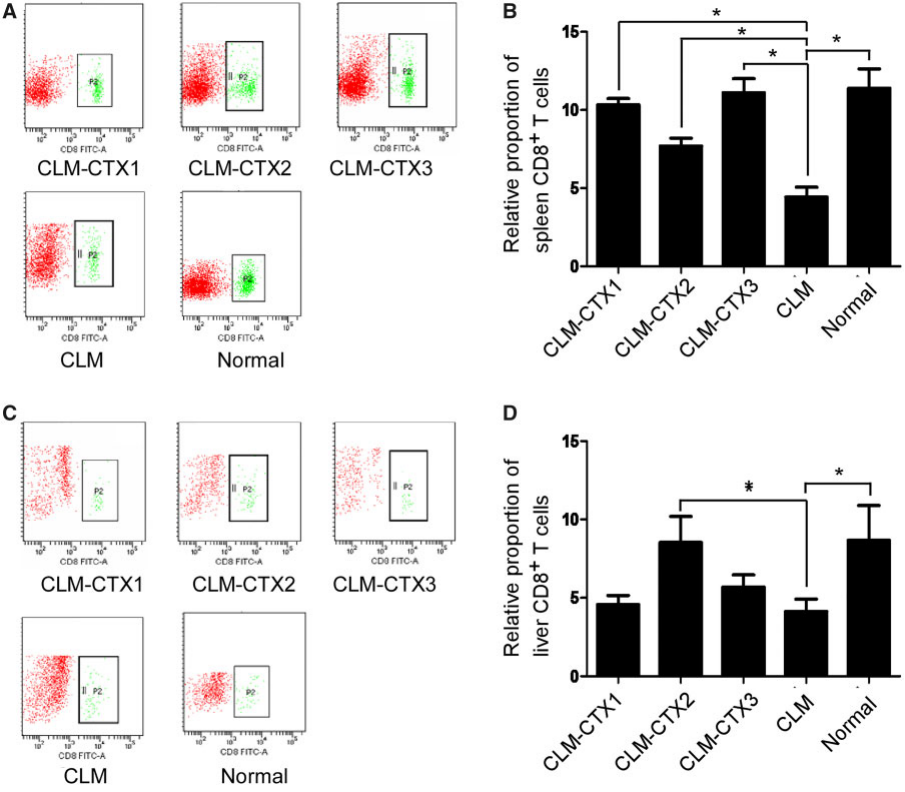
Flow-cytometry analysis of the CD8^+^ T-cell population in the spleen and liver. The number of CD8^+^ T cells in the spleen was significantly lower in the CLM group than in the other groups (*P* < 0.001), with no significant differences among the CLM-CTX1, CLM-CTX2, CLM-CTX3, and control groups (both *P* > 0.05) ((**A**) and (**B**)). In the liver, the number of CD8^+^ T cells significantly decreased in the CLM group compared with that in the CLM-CTX2 (*P* = 0.020) and control groups (*P* = 0.017). No significant differences were observed among the CLM, CLM-CTX1, and CLM-CTX3 groups (*P* = 0.690 and *P* = 0.327) ((C) and (D)). Mice in the CLM-CTX1 group (*n* = 6) were intraperitoneally injected with CTX (20 mg/kg) post surgery immediately. Mice in the CLM-CTX2 group (*n* = 6) were intraperitoneally injected with CTX (20 mg/kg) on Days 0 and 7 post surgery. Mice in the CLM-CTX3 group (*n* = 6) were intraperitoneally injected with CTX (20 mg/kg) on Days 0, 7, and 14 post surgery. The mice of the liver-metastasis-only (CLM) group (*n* = 6) were not injected with CTX. CLM, colon-cancer liver metastasis; CLM-CTX, colon-cancer liver metastasis-cyclophosphamide; CTX, cyclophosphamide.

In this study, the number of CD8^+^ T cells in the CLM group decreased after injection with CT26 cells. The numbers of CD8^+^ T cells in the spleens of mice injected with CT26 cells were significantly lower in the CLM group than those in the other groups (*P* < 0.001), with no significant differences among the CLM-CTX1, CLM-CTX2, CLM-CTX3, and control groups (both *P* > 0.05) (Figure 4A and B). A similar trend was observed in the liver; that is, the number of CD8^+^ T cells significantly decreased in the CLM group compared with those in the CLM-CTX2 (*P* = 0.020) and control groups (*P* = 0.017). However, no significant differences were observed among the CLM, CLM-CTX1, and CLM-CTX3 groups (*P* = 0.690 and *P* = 0.327) (Figure 4C and D).

### Flow-cytometry analysis of the CD4^+^ T-cell population

**Figure 5.**
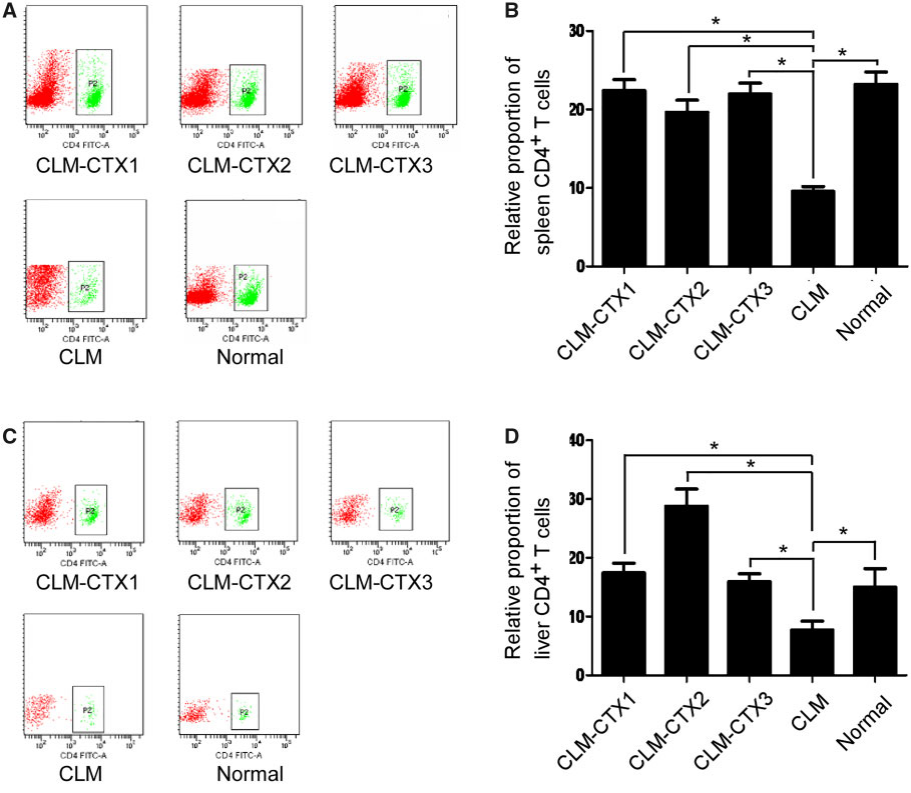
Flow-cytometry analysis of the CD4^+^ T-cell population in the spleen and liver. In the spleen, the number of CD4^+^ T cells significantly decreased in the CLM group (*P* < 0.001) and there were no significant differences among the CLM-CTX1, CLM-CTX2, CLM-CTX3, and control groups (both *P* > 0.05) ((**A**) and (**B**)). A similar trend was also observed in the liver (*P* < 0.001) ((**C**) and (**D**)). Mice in the CLM-CTX1 group (*n* = 6) were intraperitoneally injected with CTX (20 mg/kg) post surgery immediately. Mice in the CLM-CTX2 group (*n* = 6) were intraperitoneally injected with CTX (20 mg/kg) on Days 0 and 7 post surgery. Mice in the CLM-CTX3 group (*n* = 6) were intraperitoneally injected with CTX (20 mg/kg) on Days 0, 7, and 14 post surgery. The mice of the liver-metastasis-only (CLM) group (*n* = 6) were not injected with CTX. CLM, colon-cancer liver metastasis; CLM-CTX, colon-cancer liver metastasis-cyclophosphamide; CTX, cyclophosphamide.

In this study, while the number of CD4^+^ T cells significantly decreased in the spleens of the CLM-group mice (*P* < 0.001), there were no significant differences among the CLM-CTX1, CLM-CTX2, CLM-CTX3, and control groups (both *P* > 0.05) (Figure 5A and B). A similar trend was also observed in the liver (*P* < 0.001) (Figure 5C and D).

### Expression of IL-10 and TGF-β1 in liver metastasis from colon cancer

**Figure 6.**
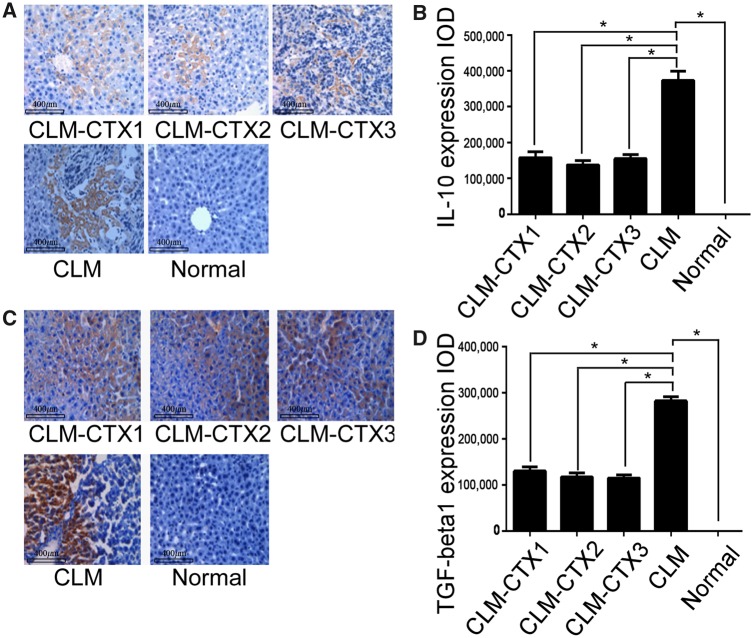
Expression of IL-10 and TGF-β1 in the liver metastases of colon cancer. The expression of IL-10 was negative in the liver tissues of normal mice and extremely high in the CLM group compared with that in the three CLM-CTX groups (*P* < 0.001) ((**A**) and (**B**)). A similar trend was observed with respect to the TGF-β1 expression in the liver tissues (*P* < 0.001) ((**C**) and (**D**)). Mice in the CLM-CTX1 group (*n* = 6) were intraperitoneally injected with CTX (20 mg/kg) post surgery immediately. Mice in the CLM-CTX2 group (*n* = 6) were intraperitoneally injected with CTX (20 mg/kg) on Days 0 and 7 post surgery. Mice in the CLM-CTX3 group (*n* = 6) were intraperitoneally injected with CTX (20 mg/kg) on Days 0, 7, and 14 post surgery. The mice of the liver-metastasis-only (CLM) group (*n* = 6) were not injected with CTX. CLM, colon-cancer liver metastasis; CLM-CTX, colon-cancer liver metastasis-cyclophosphamide; CTX, cyclophosphamide; IOD, integrated optical density.

We investigated the expression of IL-10 and TGF-β1 in liver tissue during liver metastasis of colon cancer. IL-10 and TGF-β1 were not expressed in the liver tissues of normal mice and expression levels were much higher in the CLM group than in the three CLM-CTX groups (both *P* < 0.001) (Figure 6).

### Expression of IL-10 and IFN-γ in the serum of metastasis-induced mice

**Figure 7.**
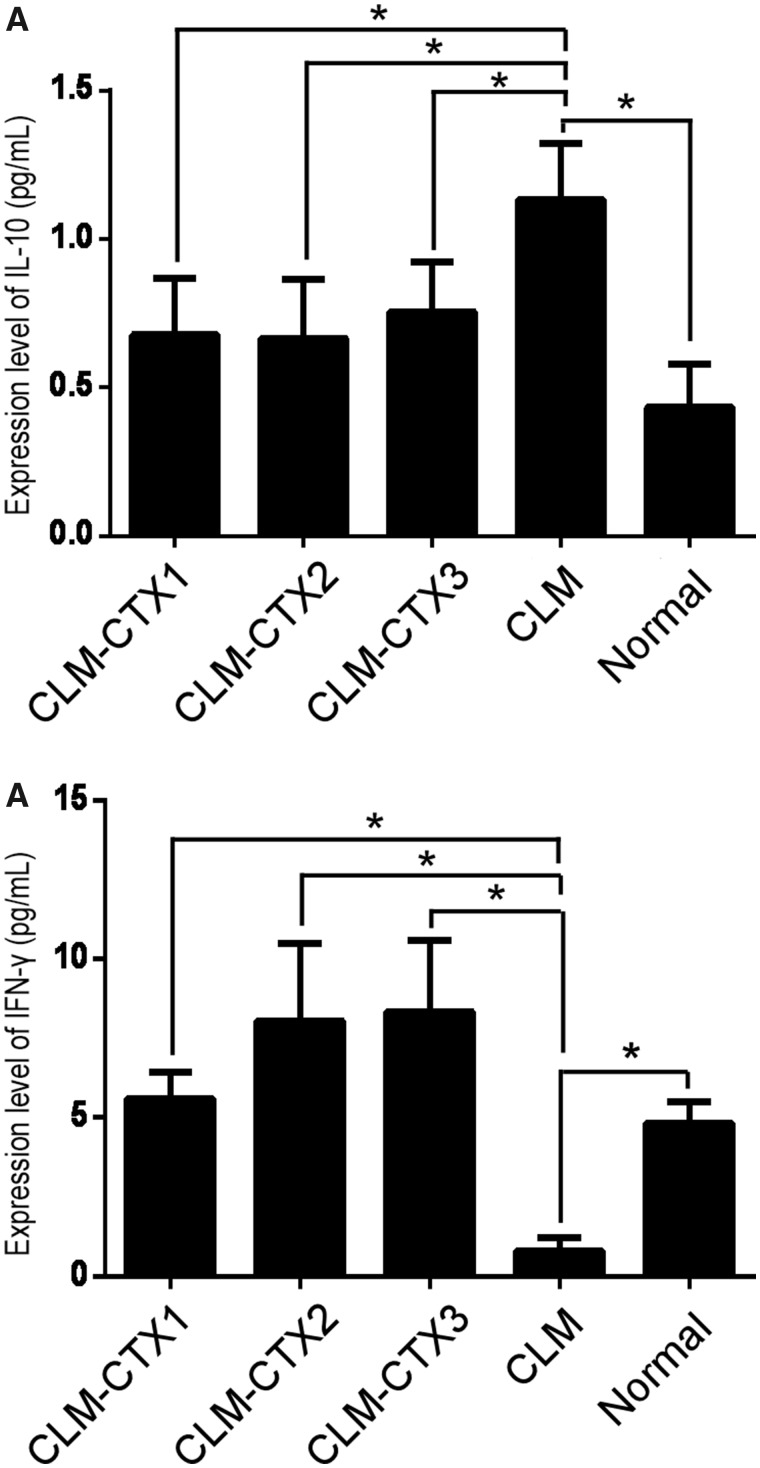
Expression of IL-10 and IFN-γ in the serum of metastasis-induced mice. IL-10 levels in the CLM group were significantly higher than those in other groups (*P* = 0.032). No differences in IL-10 levels were observed among the CLM-CTX and control groups (both *P* > 0.05) (**A**). The IFN-γ levels in the CLM group were significantly lower than those in the other groups (*P* = 0.015) (**B**). Mice in the CLM-CTX1 group (*n* = 6) were intraperitoneally injected with CTX (20 mg/kg) post surgery immediately. Mice in the CLM-CTX2 group (*n* = 6) were intraperitoneally injected with CTX (20 mg/kg) on Days 0 and 7 post surgery. Mice in the CLM-CTX3 group (*n* = 6) were intraperitoneally injected with CTX (20 mg/kg) on Days 0, 7, and 14 post surgery. The mice of the liver-metastasis-only (CLM) group (*n* = 6) were not injected with CTX. CLM, colon-cancer liver metastasis; CLM-CTX, colon-cancer liver metastasis-cyclophosphamide; CTX, cyclophosphamide.

Serum IL-10 levels were significantly higher in the CLM group than in the other groups (*P* = 0.032). There were no differences in IL-10 levels between the CLM-CTX and control groups (both *P* > 0.05) (Figure 7A). IFN-γ levels were significantly lower in the CLM group than in the other groups (*P* = 0.015) (Figure 7B).

### Survival time of mice with liver metastases from colon cancer

**Figure 8.**
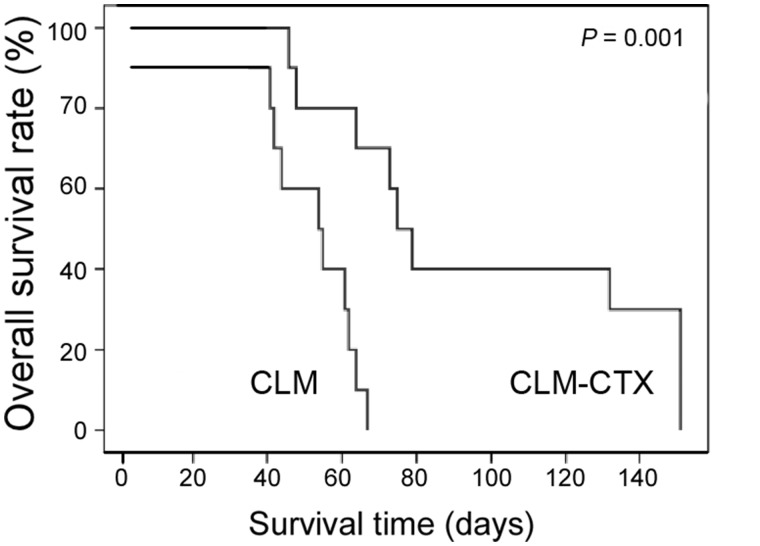
Survival time of mice with liver metastases of colon cancer. The survival time of mice in the CLM-CTX group (*n* = 10) was significantly longer than that of mice in the CLM group (*n* = 10) (*P* = 0.001). Mice of the CLM-CTX group were injected with low-dose CTX (20 mg/kg) into the abdomen on the day of surgery to establish liver metastasis of colon cancer, while the CLM-group mice were injected with the same amount of saline. CLM, colon-cancer liver metastasis; CLM-CTX, colon-cancer liver metastasis-cyclophosphamide; CTX, cyclophosphamide.

Liver metastasis from colon cancer was established in two groups of mice to investigate the effect of CTX treatment on survival. Mice in the CLM-CTX group were injected with low-dose CTX into the abdomen on the day of surgery, while the CLM-group mice were injected with the same amount of saline. The survival times were recorded and a survival curve was plotted. The survival time of mice in the CLM-CTX group was significantly longer than that of mice in the CLM group (*P* = 0.001) (Figure 8). No tumors were observed in either the spleens or livers of three mice in the CLM-CTX group after sacrifice at 149 days post surgery.

## Discussion

Our results clearly demonstrated that low-dose CTX selectively suppressed the number of IL-10- and TGF-β1-positive cells, increased the number of conventional CD4^+^ and CD8^+^ T cells in the spleen, and efficiently inhibited liver metastasis of murine CT26 colon-cancer cells. These findings may lead to an important strategy for the prevention of cancer metastasis and provide a background for the application of a combination of immunotherapy and chemotherapy for the treatment of cancer.

Liver metastasis is the main cause of death in patients with colorectal cancer. Several studies have indicated that the immune microenvironment is one of the most important prognostic factors of liver metastasis from colorectal cancer [23–25]. Infiltrating immune cells are increasingly accepted as generic constituents of tumors. These inflammatory cells operate in conflicting ways, with both tumor-antagonizing and tumor-promoting leukocytes found in various proportions in most, if not all, neoplastic lesions [26]. Antigen-sensitized cytotoxic CD8^+^ T cells are particularly important effector cells for antitumor immune responses, while CD4^+^ T cells can induce immunostimulatory or immunosuppressive effects. Specific helper T cells are required for the functions of cytotoxic T cells (CTLs) because they release cytokines that serve as effectors of different CTL-mediated tumor immune responses. T helper type 1 (Th1) CD4^+^ T cells can secrete cytokines such as IFN-γ to activate antigen-presenting cells, which in turn activate CTLs to kill tumor cells [27]. Th2 CD4^+^ T cells can suppress the activation of antigen-presenting cells and enhance humoral immunity and immune reactions [28]. CD4^+^CD25^+^FOXP3^+^ T cells can suppress the antitumor immune responses of T cells via the secretion of anti-inflammatory factors such as IL-10 and TGF-β1 [29]. IL-10, an anti-inflammatory cytokine that is overexpressed in colon cancer, plays an important role in the progression and metastasis of colon cancer and is considered to be an independent prognostic biomarker of surgical results and recurrences [30]. TGF-β is a secretory ligand that plays an important role in the initiation, development, and metastasis of tumors [31]. It suppresses systemic immunity and host immune surveillance mechanisms. The antitumor immune responses of CD8^+^ T cells can be enhanced by the removal of TGF-β [32]. Previous studies have suggested that a high density of memory CD8^+^ T cells could be an independent prognostic biomarker for improved overall survival in colon-cancer patients [33]. Moreover, an increase in Th1 CD4^+^ T-cell expression can reduce the risk of recurrences of colon cancer [34]. Injections of tumor-specific T cells into solid tumors in patients have been shown to be successful in many cases. Dudley *et al*. reported that injections of autologous T cells extracted from tumor infiltrating lymphocytes into patients with refractory melanoma improved the condition of 51% of the 35 patients [35]. However, a large number of tumor cells still escape immune surveillance.

Tumor cells escape immune surveillance via numerous mechanisms, such as via tumor-associated immunoproteins and antigens. However, systemic immunotolerance is the most important mechanism used by tumor cells to escape immune surveillance [36]. Previous studies have shown that Treg cells suppress inflammation and autoimmunity, which play important roles in the inhibition of antitumor immune responses. Tumor cells can create an inhibitory local microenvironment that directly affects the functioning of infiltrating immune cells. For example, tumor cells can down-regulate the expression of many factors, such as the major histocompatibility complex molecules, during the antigen-presenting process. They can also secrete many immunosuppressive factors, such as IL-10 and TGF-β. Moreover, tumor cells can gather Treg cells and inhibit the maturation of dendritic cells [37] and express ligands that interact with infiltrating T cells to inhibit or weaken the functions of specific CTLs [38, 39]. The tumor stroma also plays a role in the inhibition of antitumor immune responses; research has shown that it can secrete cytokines and other soluble factors that promote tumor growth and remodeling as well as inhibit the functions of T cells [40]. In this study, the number of CD4^+^ and CD8^+^ T cells in the liver and spleen significantly decreased in the CLM group compared with those in the normal group, which showed that the cellular immunity of the liver and spleen decreased during the development of liver metastases from colon cancers. Therefore, we speculate that a failure of cellular immune responses may be the main reason for liver metastasis from colon cancer.

Immunomodulation by CTX is due to cytotoxic intermediates that initiate a complex network of events, including the depletion of susceptible immune cells, attenuation of the function of more resilient immune cells, increased immunogenicity of tumor cells, and systemic effects that influence immune function [41]. Studies have indicated that the main functions of Treg cells include the maintenance of immune tolerance and suppression of autoimmune reactions. Furthermore, it has been suggested that patient prognosis gradually worsens with increases in the proportion of Treg cells in the body [41]. Treg cells have been reported to be overexpressed in many malignant tumors, including breast, ovarian, non-small-cell lung, and pancreatic cancers [42]. Because low-dose CTX can reduce the number of Treg cells in tumor-burdened mice, the regulation of immunity exerted by low-dose CTX may be due to a reduction in the number of Treg cells [43, 44]. In our study, we observed that the proportions of CD4^+^CD25^+^FOXP3^+^ Treg cells in the total CD4^+^ T-cell populations in livers were significantly higher in the CLM group than in the other groups. Thus, low-dose CTX could significantly reduce the proportion of CD4^+^CD25^+^FOXP3^+^ Treg cells and decrease the expression of IL-10 and TGF-β1 in the liver. However, no significant differences were observed among the five groups with respect to the proportion of CD4^+^CD25^+^FOXP3^+^ Treg cells in the spleen, which may be because the effect of low-dose CTX is more evident in local tumor microenvironments.

Although high-dose CTX is highly cytotoxic and leads to immunosuppression, low-dose CTX can promote immune regulation and enable T cells to exert their antitumor effects [13, 19, 21]. The present study showed that, compared with untreated tumor-burdened mice, the numbers of CD4^+^ and CD8^+^ T cells in the spleen and liver increased significantly after treatment with low-dose CTX. Several studies have shown that CD4^+^ and CD8^+^ T cells play major roles in antitumor immune responses and low-dose CTX can enhance T-cell immunity and improve antitumor immunity.

Previous studies have shown that low-dose CTX might change the cytokine-secretion profile of helper T cells from a Th2 profile typified by the secretion of IL-4 and IL-10 to a Th1 profile that has increased secretion of type 1 interferons and IL-2 [45, 46]. By secreting IL-2, Th1 cells enable the proliferation and expansion of memory CD8^+^ CTLs required for tumor-cell lysis [45]. An increase in IL-17-producing CD4^+^ T cells indicative of Th17 polarization has also been observed following low-dose CTX treatment [46]. Th17 cells are polyfunctional in cancer, secreting IL-2, tumor necrosis factor, and IFN-γ [47, 48]. In the present study, immunohistochemical staining indicated that IL-10 and TGF-β1 were overexpressed in the livers of the CLM-group mice, whereas they were not expressed in normal mice. Furthermore, our previous research indicated that the expression of IL-10 and TGF-β1 increases with an increase in the number of colon-cancer cells in the circulation. Moreover, the serum levels of IFN-γ, a pro-inflammatory factor that exerts antitumor effects and promotes immune regulation to enhance the expression of natural killer cells, macrophages, and T cells, were significantly lower in the CLM group than in the low-dose CTX-treatment groups. These results are consistent with previous research, which suggests that low-dose CTX can increase the proliferative response of CD4^+^ and CD8^+^ T cells, reduce the proportion of Treg cells, and thus affect the secretion of cytokines.

The different effects of low-dose CTX on Treg cells and effector T-cell populations may be due to the loss of essential subcellular machinery required for drug detoxification from Treg cells. Treg cells lack the expression of the ATP-binding cassette (ABC) transporter B1, which allows the efflux of CTX metabolites [49]. This difference in the subcellular machinery between Treg cells and effector T cells, which makes Treg cells more susceptible to depletion, is also evident in nuclei. Another study showed that, compared to effector T-cell populations, Treg cells have a defect in the DNA-repair pathways. Following *in vitro* exposure of isolated Treg cells and effector T-cell populations, all cells showed increases in DNA inter-strand cross-links, yet, after 24 h, DNA inter-strand cross-links were reduced in effector-cell populations but not in Treg cells [50].

This study showed that the expression of CD4^+^ T cells, CD8^+^ T cells, and IFN-γ was down-regulated, while that of IL-10 and TGF-β1 was up-regulated in liver metastases from colon cancers in mice. Furthermore, the local and systemic microenvironments of the liver were changed, which led to reduced antitumor immune responses and thus liver metastases. However, treatment with low-dose CTX reversed the observed effects. In addition, the survival times of mice treated with low-dose CTX were significantly longer than those in the other groups. Therefore, low-dose CTX exerted its antitumor activity by changing the systemic and local immune microenvironments and enhancing immune regulation in mice. However, this study has certain shortcomings. No obvious differences were observed in the mice at different CTX-dosing frequencies, which may be due to the time- and dose-dependency of the immunomodulatory effects of low-dose CTX. However, the lack of difference in effects among the different treatment groups also suggests that a short-duration treatment may limit toxicity. Hence, the best delivery time and dosing frequency of low-dose CTX must be determined in further study.

In conclusion, low-dose CTX can enhance the immunomodulatory effects and suppress the progression of liver metastasis from colon cancer, thus providing a theoretical basis for future clinical applications of immunotherapy for cancer.

## Authors’ contributions

P.L., Y.L.W., and X.M.H. conceived of and designed the project. X.M.H., N.R.Z., and X.T.L. collected the data. X.M.H., N.R.Z., X.T.L., C.Y.Z., Y.F.Z., W.J.W., X.S.H., and W.W.H. analysed and interpreted the data. N.R.Z. and X.T.L. drafted the manuscript. All authors read and approved the final manuscript.

## Funding

This work was supported by National Key Clinical Discipline, the Fundamental Research Funds for the young teacher training program of Sun Yat-sen University [grant number 18ykpy02], Medical Scientific Research Foundation of Guangdong Province of China [grant number A2016198], and ‘5010 Clinical Research Programme’ of Sun Yat-sen University [grant number 2010012].
